# Enhanced Osseointegration Capability of Poly(ether ether ketone) via Combined Phosphate and Calcium Surface-Functionalization

**DOI:** 10.3390/ijms21010198

**Published:** 2019-12-27

**Authors:** Sunarso, Akira Tsuchiya, Riki Toita, Kanji Tsuru, Kunio Ishikawa

**Affiliations:** 1 Department of Biomaterials, Faculty of Dental Science, Kyushu University, 3-1-1 Maidashi, Higashi-ku, Fukuoka 812-8582, Japan; sunarso02@ui.ac.id (S.); tsuchiya@dent.kyushu-u.ac.jp (A.T.); tsuruk@college.fdcnet.ac.jp (K.T.); ishikawa@dent.kyushu-u.ac.jp (K.I.); 2 Department of Dental Materials, Faculty of Dentistry, Universitas Indonesia, Jalan Salemba Raya No. 4, Jakarta 10430, Indonesia; 3 Biomedical Research Institute, National Institute of Advanced Industrial Science and Technology (AIST), 1-8-31 Midorigaoka, Ikeda, Osaka 563-8577, Japan; 4 Section of Bioengineering, Department of Dental Engineering, Fukuoka Dental College, 2-15-1 Tamura, Sawara, Fukuoka 814-0193, Japan

**Keywords:** poly(ether ether ketone), osseointegration, surface modification, phosphate, calcium

## Abstract

Biomedical applications of poly(ether ether ketone) (PEEK) are hindered by its inherent bioinertness and lack of osseointegration capability. In the present study, to enhance osteogenic activity and, hence, the osseointegration capability of PEEK, we proposed a strategy of combined phosphate and calcium surface-functionalization, in which ozone-gas treatment and wet chemistry were used for introduction of hydroxyl groups and modification of phosphate and/or calcium, respectively. Surface functionalization significantly elevated the surface hydrophilicity without changing the surface roughness or topography. The cell study demonstrated that immobilization of phosphate or calcium increased the osteogenesis of rat mesenchymal stem cells compared with bare PEEK, including cell proliferation, alkaline phosphatase activity, and bone-like nodule formation. Interestingly, further enhancement was observed for samples co-immobilized with phosphate and calcium. Furthermore, in the animal study, phosphate and calcium co-functionalized PEEK demonstrated significantly enhanced osseointegration, as revealed by a greater direct bone-to-implant contact ratio and bond strength between the bone and implant than unfunctionalized and phosphate-functionalized PEEK, which paves the way for the orthopedic and dental application of PEEK.

## 1. Introduction

Poly(ether ether ketone) (PEEK) has attracted increasing attention as a prime candidate for orthopedic and dental implants due to its distinct advantages over metallic implants, including similar mechanical strength to bone, lack of a metal allergy, radiolucency, excellent chemical and sterilization resistance, and easier manufacturing [1,2,3]. Nevertheless, the highly hydrophobic, bioinert surface of PEEK leads to (1) attenuated osteoblasts and their precursor cell proliferation and osteogenic differentiation, (2) fibrous tissue penetration surrounding an implant (foreign body reaction), and (3) lack of direct bone-to-implant contact (osseointegration) and, hence, implant failure, which hampers clinical application [1,2,3,4,5]. Thus, the development of a technique to endow an osseointegration capability to PEEK implants has been research topic for orthopedic and dental applications.

Surface properties, including surface chemistry and topography, are important factors that affect cell and tissue responses to materials [6,7], and techniques for surface modification have been introduced to improve the osseointegration capability of PEEK. Coating of bioactive materials, such as hydroxyapatite, octacalcium phosphate, titanium, or titanium dioxide, on the PEEK surface markedly elevates the osseointegration capability [8,9,10,11]. However, a coating might shed from implants due to weak physical bonding, and the resulting particle debris can lead to implant loosening through osteoclast-mediated bone resorption [1,12,13]. Alternatively, modification of the surface topography by lithography, pattern transfer, sandblasting, acid-etching, or plasma immersion ion implantation, modulates cell and tissue responses through integrin-, focal adhesion-, and actin reorganization-mediated mechanotransduction signals [7,14,15,16,17,18]. We and other research groups have verified that micro-topographical PEEK surfaces prepared by sandblasting [14,18] or sulfuric-acid-etching [16] elevate cellular osteogenic differentiation and osseointegration capability. Moreover, in contrast to a smooth surface, micro-topographical surfaces provide spaces in which bone tissues can infiltrate and grow to enhance the stability of implant fixation into bone tissue. Surface chemical modification using wet-chemistry was demonstrated to enhance bioactivity of implants. Notably, we and other research groups reported that surface modification of bioactive elements and functional groups, including calcium, zinc, magnesium, strontium, and phosphate, elevate the biological performance of implants [18,19,20,21,22,23,24,25], whose osseointegration capabilities were further augmented if combined with surface topographical modification [18]. Mechanistically, these elements and functional groups are recognized by the corresponding cell membrane-bound receptors, including a family of phosphate transporters (PiT) [26,27], a calcium-sensing receptor (CaSR) [28,29], a melastatin-like transient receptor potential 7 [30], and a zinc sensing receptor/GPR39 [31], resulting in enhanced cellular responses. Interestingly, double stimulation of sensing proteins for phosphate and calcium has been proved to exert greater cell proliferation and osteogenesis than single stimulation [32,33,34,35]. As a result, the combined phosphate and calcium surface functionalization on PEEK may increase the bioactivity compared with phosphate or calcium surface functionalization alone. However, the biological performance of a phosphate and calcium co-functionalized PEEK implant has not been clarified.

The aim of this study is to explore whether combined phosphate and calcium surface-functionalization could enhance the osseointegration capability of PEEK with a micro-roughness. A micro-roughened surface was generated by sandblasting. Phosphate- and/or calcium-modified PEEK was prepared by ozone gas treatment and subsequent wet chemistry. The surfaces were characterized using X-ray photoelectron spectroscopy (XPS), scanning electron microscopy (SEM), a surface roughness tester, and contact angle meter. The in vitro bioactivity of functionalized PEEK was compared using mesenchymal stem cells (MSCs) with respect to cell proliferation, alkaline phosphatase (ALP) activity, and bone-like nodule formation. Furthermore, the in vivo osseointegration capability of PEEK was evaluated by implantation into a rat bone marrow cavity.

## 2. Results

### 2.1. Surface Modification and Characterization

**Figure 1:**
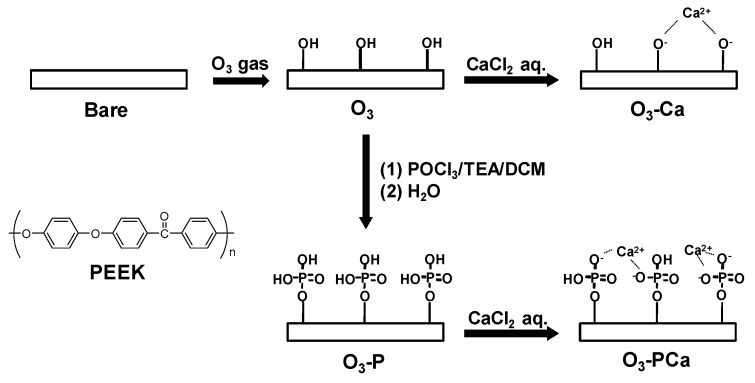
Schematic representation of the combined phosphate and calcium surface functionalization. POCl_3_: phosphoryl chloride, TEA: triethylamine, DCM: dichloromethane, PEEK: Poly(ether ether ketone).

**Figure 2:**
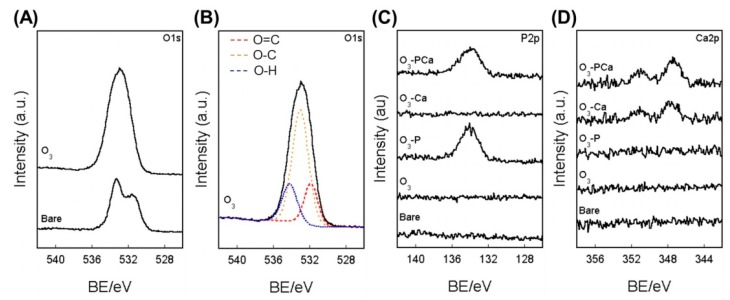
XPS analyses. (**A**) O1s spectra, (**B**) O1s peak deconvolution of O_3_, (**C**) P2p, and (**D**) Ca2p spectra. BE: binding energy, Bare: untreated PEEK, O_3_: ozone-gas-treated PEEK, O_3_-P: phosphate-immobilized PEEK, O_3_-Ca: calcium-immobilized PEEK, O_3_-PCa: phosphate and calcium co-immobilized PEEK.

A schematic of the surface modification is shown in Figure 1. The surface chemistry was analyzed using XPS (Figure 2) and the atomic composition is summarized in Table 1. The O1s peak in bare PEEK consisted of two components, as demonstrated by peaks with binding energies (BE) of 531.9 and 533.0 eV ascribed for O=C and O–C bonds, respectively (Figure 2A). Ozone-gas treatment markedly increased the at% of O1s from 19.05 to 34.88, generating a new peak with a BE of 534.2 eV for the O–H bond, as observed for the O_3_ sample (Figure 2B). The resulting ozone-gas-treated sample (O_3_) was further reacted with phosphoryl chloride in the presence of triethylamine to prepare a phosphate-modified PEEK (O_3_-P). The O_3_-P sample showed a P2p peak at a BE of 134 eV, suggesting the successful modification of phosphate on PEEK (Figure 2C). To prepare phosphate and calcium co-immobilized PEEK (O_3_-PCa), the O_3_-P sample was immersed in calcium chloride aqueous solution. XPS analysis revealed that a Ca2p peak was newly developed on the O_3_-PCa sample, while a P2p peak remained (Figure 2C,D), indicating that phosphate and calcium were successfully co-immobilized on PEEK. A similar level of P2p peak was observed in the O_3_-P and O_3_-PCa samples with at% of ≈ 1.6. The calcium-modified sample (O_3_-Ca) could be prepared by immersing ozone-gas-treated PEEK in a calcium chloride aqueous solution, as revealed by Ca2p peak (Figure 2D). The O_3_-PCa and O_3_-Ca showed similar at% of Ca2p (≈0.6 at%).

**Figure 3:**
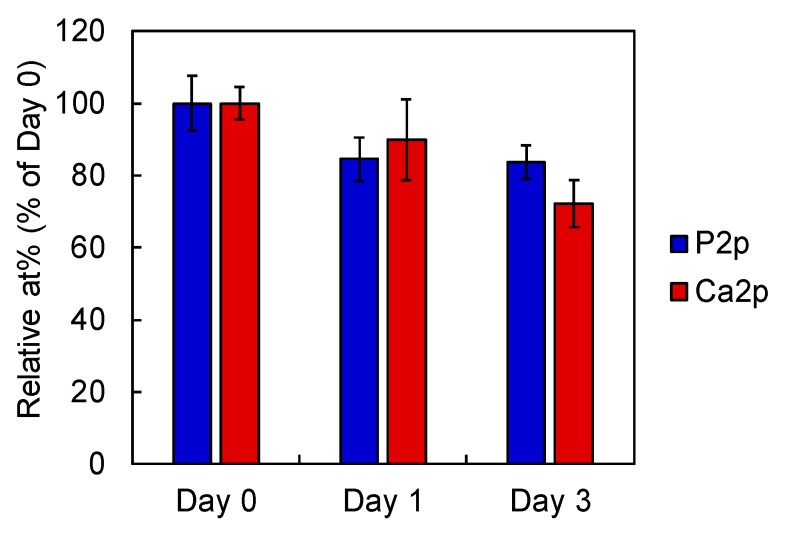
Phosphate and calcium release profile from phosphate and calcium co-immobilized PEEK after immersing in saline at 37 °C (*n* = 4). Data are the mean ± standard derivation.

**Figure 4:**
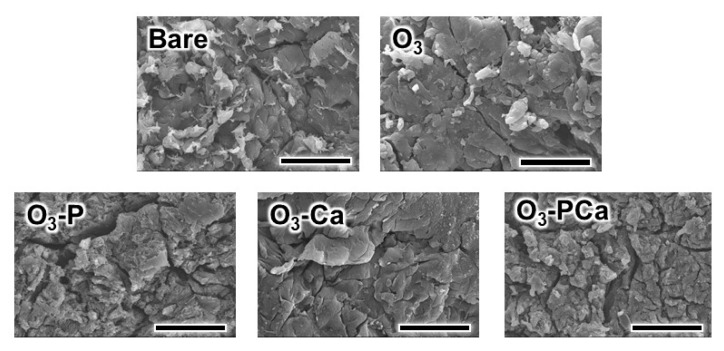
SEM images of the samples. Scale bars are 20 μm. Bare: untreated PEEK, O_3_: ozone-gas-treated PEEK, O_3_-P: phosphate-immobilized PEEK, O_3_-Ca: calcium-immobilized PEEK, O_3_-PCa: phosphate and calcium co-immobilized PEEK.

After immersing the O_3_-PCa in saline, the level of residual P2p and Ca2p slightly decreased at one and three days, indicating that phosphate and calcium were released from the O_3_-PCa (Figure 3). The SEM images indicated that the surface topography of bare and treated PEEK was similar (Figure 4). In addition, all samples showed a similar mean surface roughness (R_a_) ranging from 2.2 to 2.3 μm and mean peak to valley height (R_z_) ranging from 13.8 to 15.6 μm (Table 2). The water contact angle (WCA) of the samples was determined to estimate the surface hydrophilicity (Table 2). The relatively large WCA (≈130°) observed in the bare PEEK indicated highly hydrophobic surface. However, the WCA of the treated samples dramatically dropped owing to the hydrophilic hydroxyl and/or phosphate groups.

### 2.2. Cellular Responses

**Figure 5:**
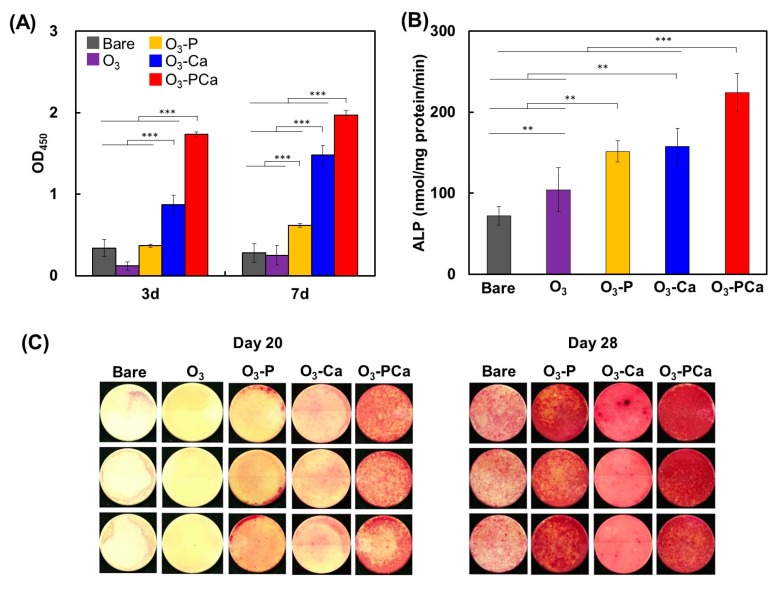
Mesenchymal stem cell responses to samples. (**A**) Cell proliferation (*n* = 4), (**B**) ALP activity at 14 days (*n* = 4), (**C**) bone-like nodule formation at 20 and 28 days (*n* = 3). Data are the mean ± standard derivation (** *p* < 0.01, *** *p* < 0.001). Bare: untreated PEEK, O_3_: ozone-gas-treated PEEK, O_3_-P: phosphate-immobilized PEEK, O_3_-Ca: calcium-immobilized PEEK, O_3_-PCa: phosphate and calcium co-immobilized PEEK.

MSCs were cultured on the samples and their cell responses, including cell proliferation, ALP activity, and bone-like nodule formation, were compared. As shown in Figure 5A, at three and seven days, the O_3_-PCa showed the greatest cell proliferation among the samples examined. The O_3_ failed to increase the cell proliferation over seven days. The O_3_-Ca displayed significantly increased cell proliferation compared with the bare PEEK sample. The O_3_-P showed a slight increase in the cell proliferation on day 7, but not on day 3. The ALP activity and bone-like nodule formation were usually used as osteogenic differentiation markers. As shown in Figure 5B, the O_3_-P and O_3_-Ca showed higher ALP activity than the bare PEEK sample. Importantly, further augmentation in the ALP activity was detected after combined phosphate and calcium surface-functionalization, as observed for the O_3_-PCa. Bone-like nodules were stained by Alizarin Red (Figure 5C). The O_3_ and bare PEEK samples failed to form bone-like nodules at 20 days. However, MSCs formed the largest mass of bone-like nodules on the O_3_-PCa sample, whereas, in accordance with the ALP activity data, a limited level of bone-like nodules was detected on the O_3_-P and O_3_-Ca samples. At 28 days, the greatest level of bone-like nodule formation was also detected from the O_3_-PCa sample. The O_3_-P showed larger bone-like nodule formation than the O_3_-Ca and bare sample, suggesting superior osteogenic activity of the O_3_-P to those of the O_3_-Ca and bare. Overall, these results indicate that the combined surface functionalization possessed greater osteogenic activity than the single surface functionalization.

### 2.3. Osseointegration Capability

Samples were inserted into the rat bone marrow cavity, and the bone-to-implant contact ratio (BIC: defined as the percentage of implant length covered by new bone), and bone-to-implant bond strength (pullout force) were determined two and four weeks after insertion. The BIC and pullout force are usually determined to quantify the osseointegration capability. Given the notable cell responses to O_3_-PCa and O_3_-P among the samples tested (Figure 5), we selected O_3_-PCa, O_3_-P, and bare (control) as test samples to reduce the number of experimental animals used.

**Figure 6:**
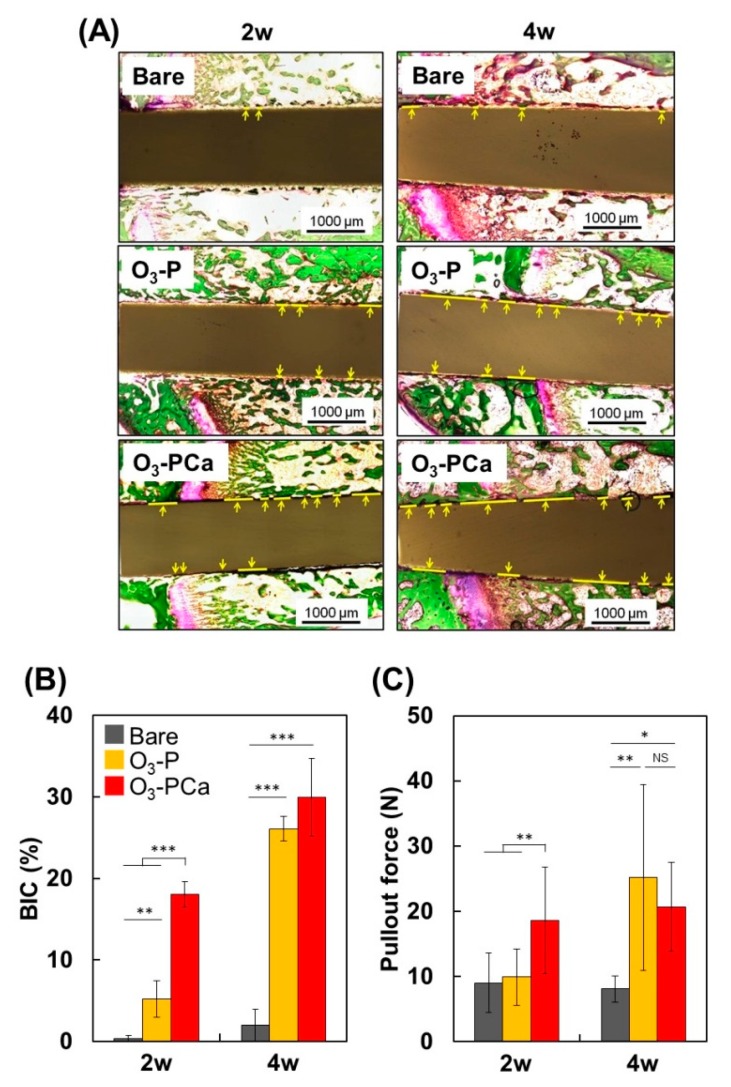
Osseointegration capability of samples in a rat femur implantation model. (**A**) Histological images of new bone formation and osseointegration at two- and four weeks post-implantation. Sections were stained by the Villanueva-Goldner stain. Yellow lines indicate bone tissue directly in contact with implants. Scale bars are 1000 μm. (**B**) Bone-to-implant contact ratio (BIC) (*n* = 3), and (**C**) bond strength between bone and implant (pullout force) (*n* = 6). Data are mean ± standard derivation (* *p* < 0.05, ** *p* < 0.01, *** *p* < 0.001, NS: not significant). Bare: untreated PEEK, O_3_-P: phosphate-immobilized PEEK, O_3_-PCa: phosphate and calcium co-immobilized PEEK.

We first prepared Villanueva-Goldner stained histological sections (Figure 6A) and calculated BIC (Figure 6B). As expected, the BIC of bare PEEK was less than 2% at two and four weeks, indicating lack of osseointegration capability of PEEK. At two weeks, the BIC of the O_3_-PCa and O_3_-P were 18% and 5%, which was approximately 50-times and 15-times greater than the bare PEEK sample (0.3%), respectively. At four weeks, both O_3_-PCa (30%) and O_3_-P (26%) significantly increased BIC, but not bare PEEK (2%), resulting in approximately much higher BIC in the O_3_-PCa and O_3_-P than the bare sample. The pullout force was then measured (Figure 6C). At two weeks, the pullout force of the O_3_-PCa was markedly higher than those of the bare and O_3_-P PEEK, contributing to much higher BIC of the O_3_-PCa. Despite a higher BIC level of O_3_-P than that of the bare PEEK, the O_3_-P failed to increase the pullout force. At four weeks, O_3_-PCa and O_3_-P exhibited increased pullout forces compared with the bare PEEK. Given the O_3_-PCa exhibited the greatest osteogenic activity in vitro, the O_3_-PCa implant might provide a more proper environment for endogenous MSCs, preosteoblasts ingrowth and osteogenic differentiation than the bare and O_3_-P PEEK implants, hence augmenting bone formation, BICs and pullout force at the earlier time point. Overall, the results indicate that combined phosphate and calcium surface functionalization significantly enhanced the osseointegration capability and bone fixation of PEEK.

## 3. Discussion

The surface topography/roughness, hydrophilicity, and chemical composition potentiate cellular responses to materials [6,7,9,36,37,38,39]. However, in the present study, all of the functionalized surfaces showed similar surface topography/roughness to those of bare PEEK, and thus can be excluded as a factor in the observed differences of MSCs behaviors on the functionalized PEEK. Alternatively, PEEK has a highly hydrophobic surface, but surfaces became more hydrophilic after surface functionalization. It is widely accepted that a hydrophilic surface produced a more pronounced effect on the cell responses than a hydrophobic surface [36,37,38,39]. Notably, the O_3_-Ca and O_3_-PCa showed enhanced cell proliferation compared with the bare at three and seven days. However, the O_3_ and O_3_-P failed to improve cell proliferation. Ozone gas functionalization and phosphate modification on PEEK produced negatively-charged hydroxyl groups and phosphate groups, respectively, but these negative charges were canceled after calcium modification. Due to negative charges on the cell surface, it is expected that the O_3_ and O_3_-P surface might electrostatistically repel cell adhesion, resulting in a lower or similar viable cell number compared with the bare at three days. In ALP and bone-like nodule formation assays, we observed that the O_3_-PCa, O_3_-Ca, and O_3_-P samples enhanced the MSC differentiation compared with the bare sample, but the O_3_ sample failed to improve cellular response, even though the WCAs of the functionalized samples were not widely different. In our recent report, MSCs also showed a negligible increase in cell proliferation and osteogenic marker expression on hydrophilic surface generated by surface plasma-treatment of PEEK when compared with those on untreated PEEK [19]. Both ozone-gas-treated and plasma-treated PEEK lack a bioactive element and functional groups such as calcium and phosphate. Thus, we assume that the surface hydrophilicity played a limited role in enhanced MSC responses on phosphate- and/or calcium-functionalized PEEK when compared with the surface chemical composition.

Upon cell-cell contact, cells adhere to each other via gap junction proteins such as connexin 43, which mediates intercellular exchange of ions, small molecules, and second messengers, and promotes osteogenic differentiation of MSCs [40]. Knockout or genetic ablation of connexin 43 dramatically attenuated ALP activity, osteopontin and osteocalcin expression, and bone-like nodule formation [41,42]. In addition, cell density has been reported to be an important factor in osteogenic differentiation [43]. When MSCs were cultured on microislands with cell density ranging from 1 to 9 cells/microisland, the ALP activity was increased in accordance with cell number, which was abrogated by inhibition of connexin 43 with 18-α-glycyrrhetinic acid. Those reports clearly demonstrated that cell-cell junction contact through connexin 43 is important for MSC osteogenic differentiation. In the present study, higher levels of cell proliferation were observed from the O_3_-PCa and O_3_-Ca than bare PEEK, indicating that the O_3_-PCa and O_3_-Ca accomplished more cell-cell contact. In agreement with cell proliferation results, the O_3_-PCa and O_3_-Ca also showed enhanced ALP activity and bone-like nodule formation compared with bare PEEK. Thus, enhanced cell proliferation on the O_3_-PCa and O_3_-Ca could be one reason for the enhanced osteogenic differentiation on their surfaces.

Of relevance to bone forming cells and MSCs, phosphate and calcium are not only components of bone tissue but also play pivotal roles in cellular proliferation, osteogenic differentiation, and chemotaxis [32,33,34,44]. Cells sense extracellular phosphate and calcium through a variety of receptors, transporters, and channel proteins expressed on extracellular membranes, resulting in increased expression of genes/proteins involved in cell growth and osteogenesis, including but not limited to the cyclin family, ALP, osteopontin, osteocalcin, osterix, and runt-related transcription factor 2 [44,45,46,47,48,49,50,51,52]. Notably, this study demonstrated that surface functionalization of PEEK with phosphate and/or calcium enhanced MSC proliferation and osteogenic differentiation compared with the bare PEEK, most likely because the cells recognized phosphate and/or calcium modified on the surface or released from the surface through the corresponding sensing proteins and promoted cell responses accordingly. Of more interest is that the combined phosphate- and calcium-surface functionalization (O_3_-PCa) exhibited much better bioactivity than those functionalized by phosphate (O_3_-P) or calcium alone (O_3_-Ca), as evidenced by the greatest cell proliferation, ALP activity, and bone-like nodule formation observed on the O_3_-PCa. For instance, in vitro studies revealed that the level of PiT, a phosphate-sensing protein, was increased in response to calcium [44,52], by which cellular sensitivity to extracellular phosphate might be increased, resulting in the promotion of cellular responses [32,33,34]. Furthermore, both phosphate and calcium cooperatively involved in level of sodium-dependent vitamin C transporter 2 (SVCT2) expression, a transporter for ascorbic acid (vitamin C) [32]. Ascorbic acid that is usually contained in an osteogenic differentiation medium plays a crucial role in osteogenic differentiation [53]. Phosphate- and calcium-mediated increases in SVCT2 expression augmented the cellular ascorbic acid uptake and hence osteogenic differentiation, which was abrogated by PiT or a calcium-channel inhibitor. Those previous reports may support our estimation that the strongest performance observed in phosphate- and calcium-surface functionalization (O_3_-PCa) was in part due to orchestration of phosphate and calcium in MSC proliferation and osteogenic differentiation.

In a rat femur implantation model, the O_3_-PCa and O_3_-P significantly increased bone-implant integration compared with bare PEEK, as evidenced by greater BIC and pullout force. For instance, the BIC of O_3_-PCa and O_3_-P increased up to 50-fold and 15-fold at two weeks (the early-stage of healing), respectively. The pullout force of O_3_-PCa was twice that of bare PEEK, but O_3_-P was similar to bare PEEK at two weeks. The pullout force observed from the O_3_-PCa at two weeks was almost equivalent to that observed from the O_3_-P at four weeks, demonstrating that the O_3_-PCa achieved bone-implant mechanical integration twice as fast as the O_3_-P. This is because of the synergistic effect of phosphate and calcium on accelerating osseointegration. However, at four weeks, a further noticeable increase in pullout force was not detected from the O_3_-PCa, probably because of the low density of phosphate and calcium. Increasing the density of phosphate and calcium seems to have a prominent effect on increasing the osseointegration capability of PEEK. In addition, the mechanism on how phosphate and calcium co-immobilized on the PEEK surface influenced cell and tissue behavior was not investigated in this preliminary report. Hence, further study is required to fully understand how phosphate and calcium modified PEEK enhance the cellular behavior and osseointegration capability observed in this study.

## 4. Materials and Methods

### 4.1. Surface Modification

PEEK board and rod (TECAPEEK natural, Ensinger Japan, Tokyo, Japan) were machined by Sunbrain Inc. (Osaka, Japan) to obtain PEEK discs (Φ14 mm × 1 mm) and rods (Φ1.4 mm × 23 mm). PEEK discs were used for surface characterization and cell study, and rods were used for an animal study. Samples were sandblasted using alumina particles (F60) with a pressure of 0.5 MPa for 10 s (Hozan sandblast SG-106, Osaka, Japan), and ultrasonically washed with acetone and water, and dried at room temperature overnight as previously described [14]. The sandblasted samples were treated by ozone gas at room temperature for 1 h using an ozone generator (EcoDesign Inc., Saitama, Japan) at a flow rate of 2 nL/min with a current of 3.4 A to produce hydroxyl groups on the surface. The ozone-treated samples were reacted in 20 mL dichloromethane (Wako, Osaka, Japan) containing phosphoryl chloride (307 mg, 2 mmol, Wako) and triethylamine (202 mg, 2 mmol, Wako) for 24 h at room temperature. After the reaction, the samples were washed by acetone (Wako) twice, followed by deionized water for 30 min to obtain phosphate-immobilized samples. The phosphate-modified samples were immersed in a 10 mmol/L calcium chloride aqueous solution at room temperature for 30 min to obtain phosphate and calcium co-immobilized samples. The calcium-modified samples were prepared by soaking the ozone-treated samples in the 10 mmol/L calcium chloride aqueous solution at room temperature for 30 min. All treated samples were dried by flowing nitrogen gas.

### 4.2. Surface Characterization

The chemical composition of the surfaces was analyzed using XPS (K-alpha, Thermo Fisher Scientific, East Grinstead, UK). The XPS system was calibrated to give Au 4f7/2 with a BE of 83.96 eV and Ag 3d5/2 with a BE of 368.21 eV, and the maximum peaks of C 1s were set to 284.8 eV. The surface topography was observed using SEM (S-3400N, Hitachi High Technologies, Tokyo, Japan) under an accelerating voltage of 5 kV after surface coating with gold. The water contact angle (1.5 μL) on the samples was determined using a contact angle meter (DM500, Kyowa Interface Science, Saitama, Japan). The mean surface roughness (R_a_) and mean peak to valley height (R_z_) were determined using a surface roughness tester (SJ-400, Mitsutoyo, Kanagawa, Japan).

### 4.3. Cell Study

#### 4.3.1. Cell Culture

Rat MSCs were isolated from femurs of specific-pathogen-free (SPF) Wistar rats (male, four weeks old; Japan SLC, Shizuoka, Japan) and were cultured in Minimum Essential Medium α (MEMα) supplemented with 15% heat-inactivated fetal bovine serum (FBS), 100 U/mL penicillin, 100 μg/mL streptomycin, and 0.25 μg/mL amphotericin B (all from Thermo Fisher Scientific) as previously described [36].

#### 4.3.2. Cell Proliferation

MSCs were seeded at a density of 4 × 10^4^ cells/well on samples placed in a 24-well plate. At three and seven days, the cell proliferation rate was determined using the cell-counting kit (Dojindo, Kumamoto, Japan). A microplate reader (Infinite M200, TECAN, Port Melbourne, Victoria, Australia) was used to measure the optical density (OD) at 450 nm.

#### 4.3.3. ALP Activity and Bone-Like Nodule Formation

MSCs were seeded on the samples placed in a 24-well plate at a density of 4 × 10^4^ cells/well. MSCs were differentiated in MEMα supplemented with 15% heat-inactivated FBS, 100 U/mL penicillin, 100 μg/mL streptomycin, 0.25 μg/mL amphotericin B, 10 mmol/L sodium β-glycerophosphate (Wako), and 10 nmol/L dexamethasone (Wako). The medium was exchanged with fresh three times per week. At 14 days, ALP activities were determined using the Lab Assay ALP kit (Wako). Cells were washed by phosphate-buffered saline (PBS; Wako) and were lysed in cell lysis buffer M (Wako; containing 20 mmol/L Tris-HCl, 200 mmol/L sodium chloride, 2.5 mmol/L magnesium chloride, and 0.05% NP-40 substitute). Cell lysate (20 μL) and a buffer solution containing disodium p-nitrophenylphosphate (100 μL) were mixed and incubated at 37 °C for 15 min. The reaction was stopped by the addition of 80 μL of the stop solution (0.2 mol/L sodium hydroxide). The OD at 405 nm was measured using a microplate reader. The protein concentration in cell lysate was determined using the Bio-rad Protein Assay (Bio-rad Laboratories, Hercules, CA, USA). The ALP activity was normalized by the protein concentration.

At 20 days, bone-like nodules were stained using the Calcified nodule staining kit (Cosmo Bio, Tokyo, Japan). Cells were washed by PBS and fixed by methanol (Wako) cooled at −20 °C for 30 min. After washing cells with distilled water, 400 μL of the chromogenic solution containing Alizarin Red was dispensed to each well, and incubated at room temperature for 5 min. The samples were washed by buffer solution to remove unbound Alizarin Red.

### 4.4. Animal Study

Thirty SPF Wistar rats (male, 12-weeks old) were purchased from Japan SLC (Shizuoka, Japan). Rats were housed in standard cages and maintained in a temperature-controlled room (22 °C) with a 12 h light-dark cycle. Rats were fed a normal diet (CRF-1; Oriental Yeast, Tokyo, Japan) and sterilized tap water ad libitum. The animal study was performed in accordance with the Guidelines for Animal Experiments established by the National Institute of Health, the Ministry of Health, Labour, and Welfare of Japan and Kyushu University (Ethical approval number: A28-061-0).

The rats were anesthetized with intraperitoneal injection of ketamine (75 mg/kg) and xylazine (10 mg/kg). The fur surrounding the insertion site was shaved and cleaned by iodine before surgery. A 1.5 cm incision was made along the axis of the femur to open the articular capsule. A hole (Φ1.5 cm) between the condyle and bone marrow cavity parallel to the long axis of femur was made using a dental reamer. Two rod-shaped samples (Φ1.4 cm × 23 mm) were implanted bilaterally in each rat. The muscle and skin were closed by suturing using a silk suture.

At two and four weeks, the rats were sacrificed and femurs with implants were harvested for histological analysis and pullout force measurement. For histological analysis, femurs were fixed and dehydrated with a series of ethanol solution (70–100%), and then embedded in poly(methylmethacrylate) resin. A section with a thickness of 1 mm was cut using a diamond cutter and was ground to a final thickness of 60 μm using a grinding unit (Speed-lap ML-521-d, Maruto Instrument, Tokyo, Japan). The section was stained by the Villanueva-Goldner method and images were analyzed using all-in-one fluorescence microscopy (BZX710, Keyence, Osaka, Japan) to calculate the bone-to-implant contact ratio. The pullout force was determined using a universal testing machine (Autograph AGS-J, Simadzu, Kyoto, Japan) as previously described [19].

### 4.5. Statistical Analysis

The statistical significance between test groups was evaluated by one-way analysis of variance (ANOVA) followed by Fisher’s least significant difference (LSD). *p* < 0.05 was considered statistically significant.

## 5. Conclusions

We successfully prepared phosphate and/or calcium surface functionalized PEEK through ozone and chemical treatment. Combined phosphate and calcium surface-functionalization significantly elevated MSC osteogenesis in vitro and osseointegration capability in vivo compared with a single surface modification. This surface chemical functionalization is a promising technique for increasing the osseointegration capability of PEEK implants.

## Figures and Tables

**Table 1:** Chemical composition of PEEK surfaces determined by XPS analysis.

Samples	C1s/at%	O1s/at%	P2p/at%	Ca2p/at%
Bare	80.95 ± 0.33	19.05 ± 0.33	-	-
O_3_	65.12 ± 0.94	34.88 ± 0.93	-	-
O_3_-P	77.71 ± 1.44	20.65 ± 1.17	1.64 ± 0.29	-
O_3_-Ca	77.64 ± 1.39	21.77 ± 1.46	-	0.59 ± 0.12
O_3_-PCa	77.88 ± 1.53	19.99 ± 1.13	1.56 ± 0.23	0.57 ± 0.19

Data are the mean ± standard derivation. Bare: untreated PEEK, O_3_: ozone-gas-treated PEEK, O_3_-P: phosphate-immobilized PEEK, O_3_-Ca: calcium-immobilized PEEK, O_3_-PCa: phosphate and calcium co-immobilized PEEK.

**Table 2:** Surface roughness and water contact angle of PEEK surfaces.

Samples	R_a_/μm	R_z_/μm	WCA/deg.
Bare	2.3 ± 0.2	14.5 ± 1.2	133.0 ± 5.1
O_3_	2.2 ± 0.2	14.0 ± 1.2	61.5 ± 8.3
O_3_-P	2.2 ± 0.1	13.1 ± 2.2	88.9 ± 9.6
O_3_-Ca	2.2 ± 0.1	13.8 ± 1.1	85.5 ± 9.9
O_3_-PCa	2.2 ± 0.2	15.6 ± 0.7	77.9 ± 7.7

Data are the mean ± standard derivation. Bare: untreated PEEK, O_3_: ozone-gas-treated PEEK, O_3_-P: phosphate-immobilized PEEK, O_3_-Ca: calcium-immobilized PEEK, O_3_-PCa: phosphate and calcium co-immobilized PEEK, R_a_: average roughness, R_z_: mean peak to valley height, WCA: water contact angle.

## References

[1] Kurtz S.M., Devine J.N. (2007). PEEK biomaterials in trauma, orthopedic, and spinal implants. Biomaterials.

[2] Ma R., Tang T. (2014). Current strategies to improve the bioactivity of PEEK. Int. J. Mol. Sci..

[3] Buck E., Li H., Cerruti M. (2019). Surface modification strategies to improve the osseointegration of poly(etheretherketone) and its composites. Macromol. Biosci..

[4] Sagomonyants K.B., Jarman-Smith M.L., Devine J.N., Aronow M.S., Gronowicz G.A. (2008). The in vitro response of human osteoblasts to polyetheretherketone (PEEK) substrates compared to commercially pure titanium. Biomaterials.

[5] Olivares-Navarrete R., Hyzy S.L., Slosar P.J., Schneider J.M., Schwartz Z., Boyan B.D. (2015). Implant materials generate different peri-implant inflammatory factors: Poly-ether-ether-ketone promotes fibrosis and microtextured titanium promotes osteogenic factors. Spine J..

[6] Gittens R.A., Scheideler L., Rupp F., Hyzy S.L., Geis-Gerstorfer J., Schwartz Z., Boyan B.D. (2014). A review of the wettability of dental implants surface II: Biological and clinical aspects. Acta Biomater..

[7] Chen W., Shao Y., Li X., Zhao G., Fu J. (2014). Nanotopographical surfaces for stem cell fate control: Engineering mechanobiology from the bottom. Nano Today.

[8] Durham J.W., Montelongo S.A., Ong J.L., Guda T., Allen M.J., Rabiei A. (2016). Hydroxyapatite coating on PEEK implants: Biomechanical and histological study in a rabbit model. Mater. Sci. Eng. C.

[9] Kunomura S., Iwasaki Y. (2019). Immobilization of polyphosphoesters on poly(ether ether ketone) (PEEK) for facilitating mineral coating. J. Biomater. Sci. Polym. Ed..

[10] Liu S., Zhu Y., Gao H., Ge P., Ren K., Gao J., Cao Y., Han D., Zhang J. (2018). One-step fabrication of functinalized poly(etheretherketone) surfaces with enhanced biocompatibility and osteogenic activity. Mater. Sci. Eng. C.

[11] Walsh W.R., Bertollo N., Christou C., Schaffner D., Mobbs R.J. (2015). Plasma-sprayed titanium coating to polyetheretherketone improves the bone-implant interface. Spine J..

[12] Martini D., Fini M., Franchi M., Pasquale V.D., Bacchelli B., Gamberini M., Tinti A., Taddei P., Giavaresi G., Ottani V. (2003). Detachment of titanium and fluorohydroxyapatite particles in unloaded endosseous implants. Biomaterials.

[13] Tang S.M., Cheang P., Bakar M.S.A., Khor K.A., Liao K. (2004). Tension–tension fatigue behavior of hydroxyapatite reinforced polyetheretherketone composites. Int. J. Fatigue.

[14] Sunarso, Tsuchiya A., Fukuda N., Toita R., Tsuru K., Ishikawa K. (2018). Effect of micro-roughening of poly (ether ether ketone) on bone marrow derived stem cell and macrophage responses, and osseointegration. J. Biomater. Sci. Polym. Ed..

[15] Ouyang L., Chen M., Wang D., Lu T., Wang H., Meng F., Yang Y., Ma J., Yeung K.W., Liu X. (2019). Nano textured PEEK surface for enhanced osseointegration. ACS Biomater. Sci. Eng..

[16] Zhao Y., Wong H.M., Wang W., Li P., Xu Z., Chong E.Y., Yan C.H., Yeung K.W., Chu P.K. (2013). Cytocompatibility, osseointegration, and bioactivity of three-dimensional porous and nanostructured network on polyetheretherketone. Biomaterials.

[17] Ouyang L., Zhao Y., Jin G., Lu T., Li J., Qiao Y., Ning C., Zhang X., Chu P.K., Liu X. (2016). Influence of sulfur content on bone formation and antibacterial ability of sulfonated PEEK. Biomaterials.

[18] Fukuda N., Kanazawa M., Tsuru K., Tsuchiya A., Sunarso, Toita R., Mori Y., Nakashima Y., Ishikawa K. (2018). Synergistic effect of surface phosphorylation and micro-roughness on enhanced osseointegration ability of poly(ether ether ketone) in the rabbit tibia. Sci. Rep..

[19] Fukuda N., Tsuchiya A., Sunarso, Toita R., Tsuru K., Mori Y., Ishikawa K. (2019). Surface plasma treatment and phosphorylation enhance the biological performance of poly (ether ether ketone). Colloids Surf. B.

[20] Zheng Y., Liu L., Xiong C., Zhang L. (2018). Enhancement of bioactivity on modified polyetheretherketone surfaces with –COOH, –OH and –PO_4_H_2_ functional groups. Mater. Lett..

[21] Toita R., Sunarso, Rashid A.N., Tsuru K., Ishikawa K. (2015). Modulation of the osteoconductive property and immune response of poly(ether ether ketone) by modification with calcium ions. J. Mater. Chem. B.

[22] Shi X., Nakagawa M., Kawachi G., Xu L., Ishikawa K. (2012). Surface modification of titanium by hydrothermal treatment in Mg-containing solution and early osteoblast responses. J. Mater. Sci. Mater. Med..

[23] Zhang L., Ayukawa Y., Legeros R.Z., Matsuya S., Koyano K., Ishikawa K. (2010). Tissue-response to calcium-bonded titanium surface. J. Biomed. Mater. Res. A.

[24] Liu W., Li J., Cheng M., Wang Q., Yeung K.W., Chu P.K., Zhang X. (2018). Zinc-modified sulfonated polyetheretherketone surface with immunomodulatory function for guiding cell fate and bone regeneration. Adv. Sci..

[25] Wang S., Yang Y., Li Y., Shi J., Zhou J., Zhang L., Deng Y., Yang W. (2019). Strontium/adiponectin co-decoration modulates the osteogenic activity of nano-morphologic polyetheretherketone implant. Colloids Surf. B.

[26] Werner A., Dehmelt L., Nalbant P. (1998). Na^+^-dependent phosphate cotransporters: The Napi protein families. J. Exp. Biol..

[27] Biber J., Hernando N., Forster I. (2013). Phosphate transporters and their function. Annu. Rev. Physiol..

[28] Golzman D., Hendy G.N. (2015). The calcium-sensing receptors in bone-mechanistic and therapeutic insights. Nat. Rev. Endocrinol..

[29] Nielsen S.P. (2004). The biological role of strontium. Bone.

[30] Zou Z.G., Rios F.J., Montezano A.C., Touyz R.M. (2019). TRPM7, magnesium, and signaling. Int. J. Mol. Sci..

[31] Hershfinkel M. (2018). The zinc sensing receptor, ZnR/GPR39, in health and disease. Int. J. Mol. Sci..

[32] Wu X., Itoh N., Taniguchi T., Nakanishi T., Tanaka K. (2003). Requirement of calcium and phosphate ions in expression of sodium-dependent vitamin C transporter 2 and osteopontin in MC3T3-E1 osteoblastic cells. Biochim. Biophys. Acta.

[33] Khoshniat S., Bougine A., Julien M., Petit M., Rouillon T., Masson M., Gatius M., Weiss P., Guicheux J., Beck L. (2011). Phosphate-dependent stimulation of MGP and OPN expression in osteoblasts via the ERK1/2 pathway is modulated by calcium. Bone.

[34] Welldon K.J., Findlay D.M., Evdokiou A., Ormsby R.T., Atkins G.J. (2013). Calcium induces pro-anabolic effects on human primary osteoblasts associated with acquisition of mature osteocyte markers. Mol. Cell. Endocrinol..

[35] Sunarso, Toita R., Tsuru K., Ishikawa K. (2016). Immobilization of calcium and phosphate ions improves the osteoconductivity of titanium implants. Mater. Sci. Eng. C.

[36] Sunarso, Toita R., Tsuru K., Ishikawa K. (2016). A superhydrophilic titanium implant functionalized by ozone gas modulates bone marrow cell and macrophage responses. J. Mater. Sci. Mater. Med..

[37] Buser D., Broggini N., Wielamd M., Schenk R.K., Denzer A.J., Cochran D.L., Hoffmann B., Lussi A., Steinemann S.G. (2004). Enhanced bone apposition to a chemically modified SLA titanium surface. J. Dent. Res..

[38] Zhao G., Schwartz Z., Weiland M., Geis-Gerstorfer J., Cochran D.L., Boyan B.D. (2005). High surface energy enhances cell response to titanium substrate microstructure. J. Biomed. Mater. Res. A.

[39] Sunarso, Toita R., Tsuru K., Ishikawa K. (2020). Ozone-gas-mediated surface hydrophilization enhances the cell responses to titanium. Mater. Lett..

[40] Stains J.P., Watkins M.P., Grimston S.K., Hebert C., Civitelli R. (2014). Molecular mechanisms of osteoblast/osteocyte regulation by connexin43. Calcif. Tissue Int..

[41] Li Z., Zhou Z., Yellowley C.E., Donahue H.J. (1999). Inhibiting gap junctional intercellular communication alters expression of differentiation markers in osteoblastic cells. Bone.

[42] Hashida Y., Nakahama K., Shimizu K., Akiyama M., Harada K., Morita I. (2014). Communication-dependent mineralization of osteoblasts via gap junctions. Bone.

[43] Tang J., Peng R., Ding J. (2010). The regulation of stem cell differentiation by cell-cell contact on micropatterned material surfaces. Biomaterials.

[44] Lee M.N., Hwang H.S., Oh S.H., Roshanzadeh A., Kim J.W., Song J.H., Kim E.S., Koh J.T. (2018). Elevated extracellular calcium ions promote proliferation and migration of mesenchymal stem cells via increasing osteopontin expression. Exp. Mol. Med..

[45] Resende R.R., Adhikari A., Da Costa J.L., Lorencon E., Ladeira M.S., Guatimosim S., Kihara A.H., Ladeira L.O. (2010). Influence of spontaneous calcium events on cell-cycle progression in embryonal carcinoma and adult stem cells. Biochim. Biophys. Acta Mol. Cell Res..

[46] Barradas A.M., Fernandes H.A., Groen N., Chai Y.C., Schrooten J., van de Peppel J., van Leeuwen J.P., van Blitterswijk C.A., de Boer J. (2012). A calcium-induced signaling cascade leading to osteogenic differentiation of human bone marrow-derived mesenchymal stromal cells. Biomaterials.

[47] Vázquez A.G., Planell J.A., Engel E. (2014). Extracellular calcium and CaSR drive osteoinduction in mesenchymal stem cells. Acta Biomater..

[48] Ha S.W., Park J., Habib M.M., Beck G.R. (2017). Nano-hydroxyapatite stimulation of gene expression requires fgf receptor, phosphate transporter, and Erk1/2 signaling. ACS Appl. Mater. Interfaces.

[49] Beck G.R. (2003). Inorganic phosphate as a signaling molecule in osteoblast differentiation. J. Cell Biochem..

[50] Hofer A.M., Brown E.M. (2003). Extracellular calcium sensing and signaling. Nat. Rev. Mol. Cell Biol..

[51] Shih Y.R.V., Hwang Y., Phadke A., Kang H., Hwang N.S., Caro E.J., Nguyen S., Siu M., Theodorakis E.A., Gianneschi N.C. (2014). Calcium phosphate-bearing matrices induce osteogenic differentiation of stem cells through adenosine signaling. Proc. Natl. Acad. Sci. USA.

[52] Nakamura S., Matsumoto T., Sasaki J., Egusa H., Lee K.Y., Nakano T., Sohmura T., Nakahira A. (2010). Effect of calcium ion concentrations on osteogenic differentiation and hematopoietic stem cell niche-related protein expression in osteoblasts. Tissue Eng. A.

[53] Langenbach F., Handschel J. (2013). Effects of dexamethasone, ascorbic acid and β-glycerophosphate on the osteogenic differentiation of stem cells in vitro. Stem Cell Res. Ther..

